# Book Review

**Published:** 1915-12

**Authors:** 


**Tropical Diseases.** By Sir PATRICK MANSON, G.C.M.G., M.D. Fifth Edition. Pp. xxiv. 937. London: Cassell and Company, Limited. 1914. 12s. 6d. net.—In reviewing a standard text-book of front-rank authority, and now in its fifth edition, it is only necessary to mention the principal new features. These are of great interest. It is now known that Kalaazar is not confined to India, but is found also in North Africa as a children's disease, also occurring in dogs, and probably transmitted by the dog flea. A skin form of infection with the Leishman body is described from South America, called Espundia. The process of development of trypanosomes in *Glossina palpalis* is described, and the moot question of the part played by *Glossina morsitans* discussed. There is a South American trypanosome disease conveyed by a bug in Brazil. It is pointed out that antimony ought to be given as well as atoxyl in cases of trypanosomiasis. Various organisms found in relapsing fever are classified, and the value of salvarsan appreciated. New fevers are described due to the bites of the rat and of a fly called Phlebotomus (the Sand-fly). Very important is the introduction of emetine in the treatment of acute or chronic dysentery instead of ipecacuanha. The relation of beri-beri to cured white rice is set forth, and the great strides which have been made in preventing this disease. Castellani has proved that yaws is due to a spirochæte allied to *S. pallida*, and that it is readily cured by salvarsan. The English type of pellagra is described. In the Fiji Islands it appears that *Filaria nocturna* has become non-periodic because the mosquito carrier is a day-biter. These are only a few of the more important changes. It will be seen that the book has been thoroughly revised and brought right up to date.

**Index-Catalogue of the Library of the Surgeon-General's Office, United States Army.** Authors and Subjects. Second Series. Vol. XIX. U—Uzzielli. Washington: Government Printing Office. 1914. Pp. 674.—This volume contains 3,046 author titles, 3,736 subject titles of separate books and pamphlets, and 32,739 titles of articles in periodicals. "United States" occupies 70 pages, "Urine" 83 pages, and "Uterus" 292 pages of closely-printed references. The series to date includes over two million references, a statement which shows that the whole profession is under a debt of gratitude for the laborious task of the compilers of a work without which no medical library is complete.

